# Supplementary material for: Papillary Renal Cell Carcinomas Rewire Glutathione Metabolism and Are Deficient in Both Anabolic Glucose Synthesis and Oxidative Phosphorylation
Source: Cancers (Basel). 2019 Sep 3;11(9):1298. doi: 10.3390/cancers11091298 (PMC6770591; doi:10.3390/cancers11091298)
Supplement: Supplementary file 1 [file cancers-11-01298-s001.zip › cancers-570749-supplementary layout/cancers-570749-supplementary layout.docx]

# Supplementalry Materials


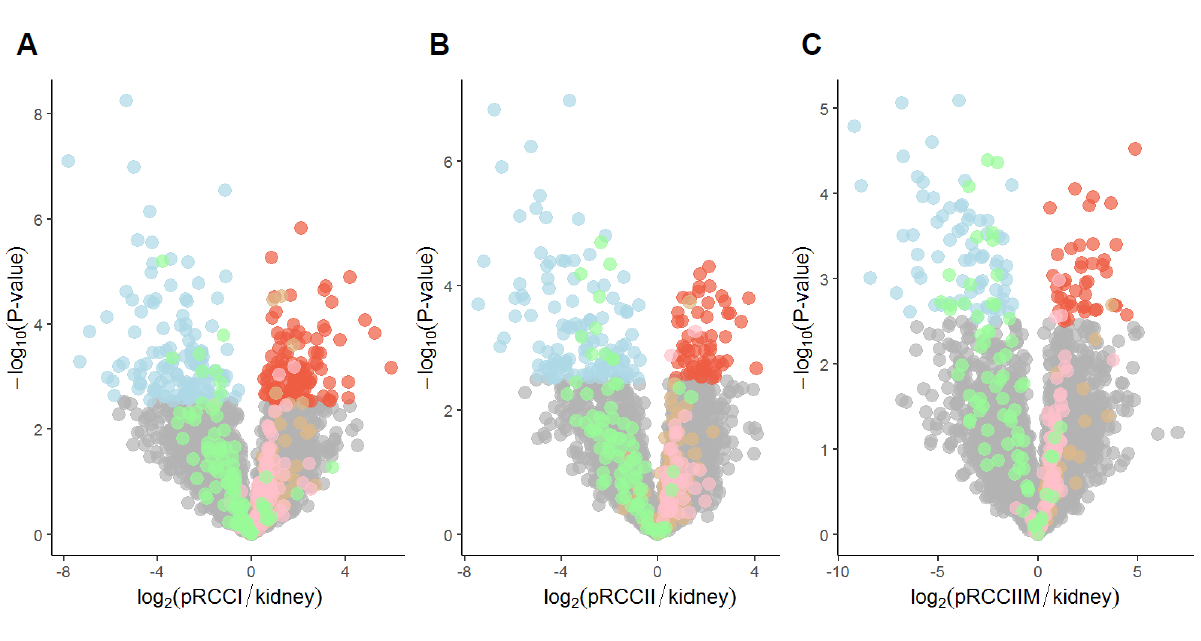


**Figure S1.** Volcano plot of log_2_ abundance ratios of (**A**) pRCC type I, (**B**) pRCC type II, and (**C**) pRCC type IIM versus kidney tissues against the -log_10_ (*p*-value) of the proteome. Indicated are following pathways: OXPHOS related metabolites in green, and ribosomes in orange. Blue, significantly decreased; red significantly increased in pRCC after considering the Benjamini-Hochberg correction (FDR of 0.05) for multiple testing. The entire list is given in Table S2.


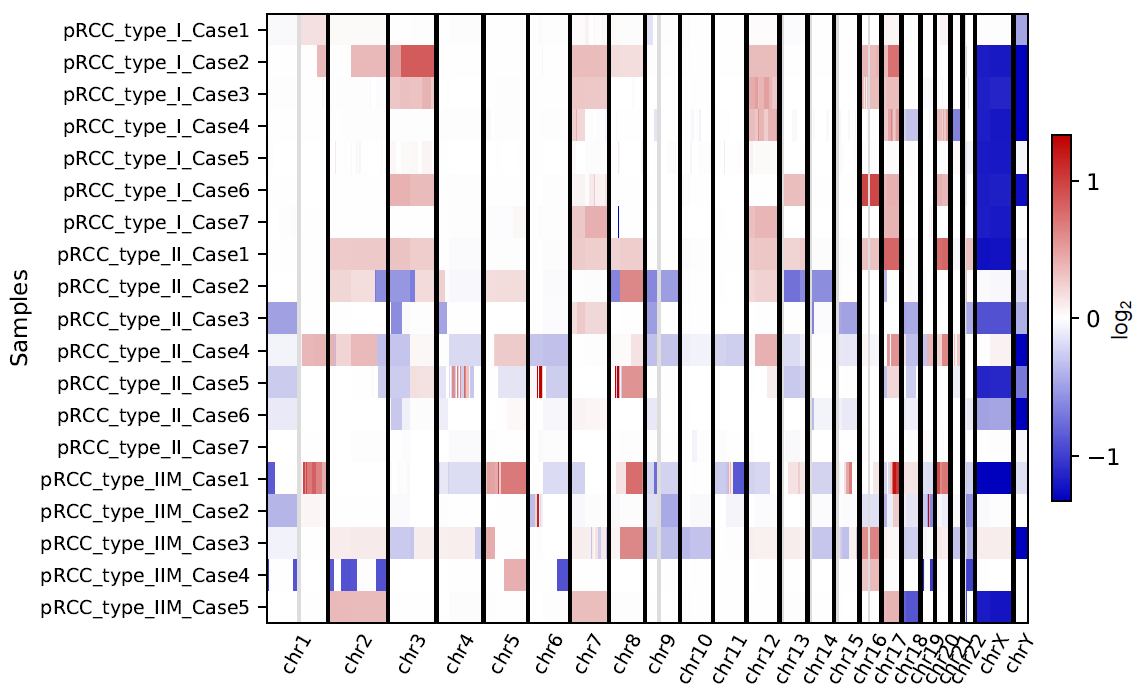


**Figure S2.** Exome-based copy number variation analysis in pRCC.


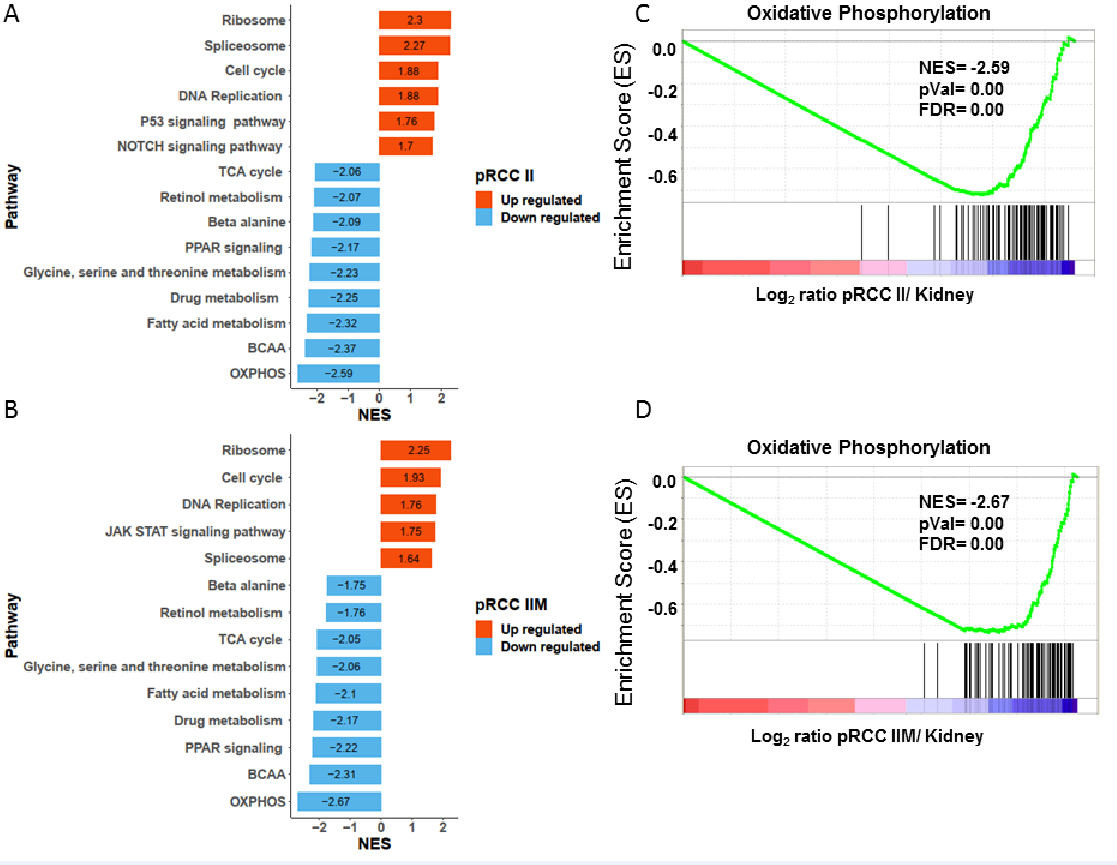


**Figure S3.** Significantly regulated KEGG pathways between the proteomes of (A) pRCC type II (**B**) pRCC type II versus healthy kidney controls. Shown is a collapsed list, the applied cutoff is *p*  ≤  0.05 and *q* ≤ 0.1. Specific enrichment plots for the KEGG pathway “oxidative phosphorylation” are shown for (**C**) the proteome of pRCC type II and (**D**) the proteome of pRCC type IIM. V-ATPases were removed from the pathway “oxidative phosphorylation” displayed in C and D, as they are wrongly assigned in this KEGG pathway. Normalized enrichment score, NES.


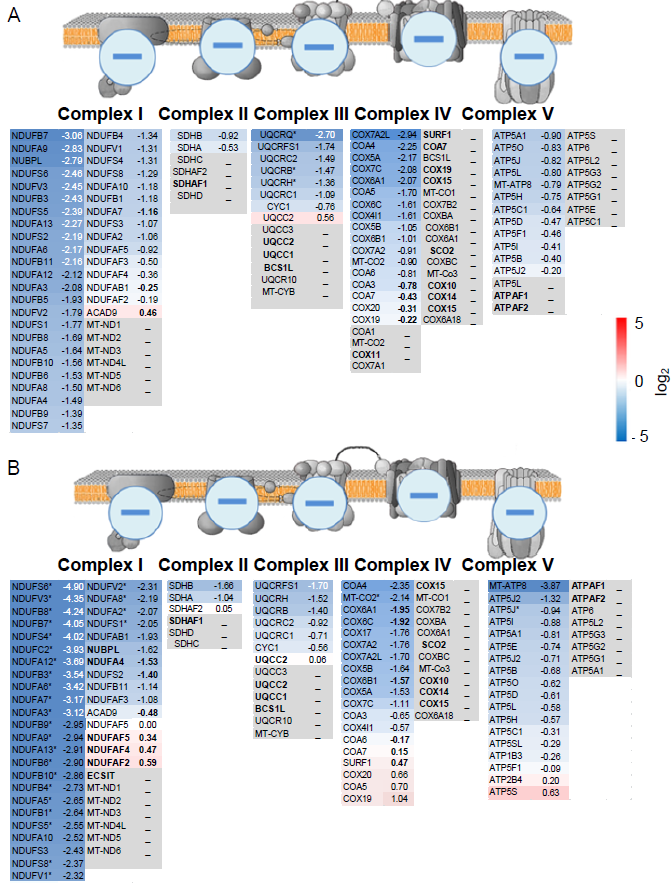


**Figure S4.** Protein abundance ratios for all individual complexes of the respiratory chain, shown for (**A**) pRCC type II and (**B**) pRCC type IIM. Illustrated are schemes of the four OXPHOS complexes and the F_0_F_1_ATPase, including subunits and assembly factors and the according log_2_ fold change between pRCC type II and pRCC type IIM versus kidney samples. The color gradient intensity in the subunit expresses the low (blue) or high (red) abundance of this protein in the tumor. Assembly factors, not part of the final complexes are shown in bold. * indicates significantly regulated proteins.

**Table S1.** MaxQuant output file featuring the proteome profiles of pRCC I, pRCC II, and pRCC IIM with LFQ intensities.

**Table S2.** Significantly regulated proteins between pRCC I, pRCC II, and pRCC IIM and healthy kidneys. List of protein groups and Log_2_ LFQ intensities identified in at least three samples of the kidney and the according pRCC type. Significance was tested with a two-sample t-test and additionally with multiple test correction by Benjamini-Hochberg (FDR 0.05), indicated by a + sign.

**Table S3.** The percentage of reconstructed genome covered by the assembly, the mean coverage depth, the number of contigs obtained and the best-predicted haplogroup are reported for each sample. TT = pRCC, NT = normal tissue, same sample numbers are derived from the same patient. The case with no mitochondrial haplogroup match between pRCC and control tissue is highlighted in yellow.

**Table S4.** Identified pathogenic somatic and germline mtDNA mutations. Indicated are variant alleles, homoplasmy, nucleotide variability, amino acid change, amino acid variability, and the disease score.

**Table S5.** Pathway enrichment analysis (GSEA) of proteins between pRCC I, pRCC II, pRCC IIM, and adjacent kidney tissues. Proteins with a minimum of three valid values in at least one group were used, missing values were replaced from normal distribution. KEGG and Reactome databases were used; the significance threshold was set to ≤0.05 for *p-value* and ≤0.10 FDR, respectively. Significant KEGG pathways were highlighted in orange.

**Table S6.** Significantly regulated transcripts between pRCC and healthy kidneys. Data were retrieved from TCGA, missing values in one group were replaced from normal distribution. Significance was tested with a two-sample t-test and additionally with multiple test correction by Benjamini-Hochberg (FDR 0.05), indicated by a + sign. Expression values are shown as log_2_ values. The TCGA sample file names encode the specimen entities as following: controls have 11 as the two last digits, pRCC type I has 01 and type II 05.

**Table S7.** Pathway enrichment analysis (GSEA) of transcriptome data between pRCC and healthy kidneys. KEGG and Reactome databases were used; the significance threshold was set to ≤0.05 for the *p-*value and ≤0.10 FDR, respectively. Significant pathways are highlighted in orange.

**Table S8.** Significantly regulated metabolites between pRCC I, pRCC II, and pRCC IIM and healthy kidneys as log_2_ peak areas. Significance was tested with a two-sample t-test and additionally with multiple test correction by Benjamini-Hochberg (FDR 0.05), indicated by a + sign. Cell culture tracing experiments to monitor glutathione and proline de novo synthesis. Shown are normalized peak areas of the first transition.

**Table S9.** Mass spectrometry settings for targeted metabolite profiling. List of all metabolites with masses, MS conditions, and MRM ion ratios for the targeted LC/MS metabolite approach. Parameters for detection were optimized with prior measurements of the pure metabolites. If the pure substance was not available, parameters were created via database information (METLIN/HMDB), in-house solutions (MS/MS) or based on values of similar chemical structure (MRM’s not tuned in). For metabolites without a detectable third transition, a pseudo-MRM, measuring the precursor mass was used. A retention time (RT) of 0 min indicates that the metabolite was measured continuously, due to large peak widths or lack of optimized elution time information. Added internal standards are shown and the standard used for normalization within the method is highlighted as such. (DP = Declustering Potential, CE = Collision Energy, CXP = Collision Exit Potential).
